# Myalgic Encephalomyelitis/Chronic Fatigue Syndrome diagnostic reporting in the 2021–2023 National Health Interview Survey

**DOI:** 10.1186/s12889-026-27598-5

**Published:** 2026-05-08

**Authors:** Katherine Fleig, Richard Nahin, Barbara Stussman, Miciah Wilkerson, Elizabeth R. Unger, Jin-Mann S. Lin, Brian Walitt

**Affiliations:** 1 National Institute of Neurological Disorders and Stroke, Bethesda, MD USA; 2 National Center for Complementary and Integrative Health, Bethesda, MD USA; 3 Centers for Disease Control and Prevention, Atlanta, GA USA

**Keywords:** Myalgic Encephalomyelitis/Chronic Fatigue Syndrome, Epidemiology, Diagnosis, Remission

## Abstract

**Background:**

Myalgic Encephalomyelitis/Chronic Fatigue Syndrome (ME/CFS) is a chronic disabling illness characterized by activity limitations associated with fatigue, post-exertional malaise (PEM), unrefreshing sleep, memory and concentration problems, orthostatic intolerance and painful discomfort. While typically considered to be a chronic condition, some persons who have had ME/CFS report no longer having the disorder. Here, the prevalence and characteristics of adults in the United States who self-report having an ME/CFS diagnosis and those who self-report no longer having ME/CFS are presented.

**Methods:**

The current study utilized publicly available data from the 2021–2023 National Health Interview Survey, which interviewed 86,655 United States civilian non-institutionalized adults about their health. For this study, participants were categorized into three groups: Current ME/CFS (individuals currently diagnosed with ME/CFS), Past ME/CFS (individuals who were previously diagnosed but no longer report having the condition), and Never ME/CFS (individuals who have never been diagnosed with ME/CFS). These groups were characterized using descriptive statistics.

**Results:**

In the United States adult population, 20.7% of the estimated 1.5% adults who ever received an ME/CFS diagnosis report they no longer have the condition (Past ME/CFS). Overall the Past ME/CFS group reported experiencing symptoms less frequently, less difficulty with daily living, approximately equal prevalence of comorbidities, and better general health status than the Current ME/CFS group but remained significantly impaired compared to the Never ME/CFS group. However, 40–50% of adults with Past ME/CFS report symptoms and function similar to adults with Current ME/CFS and only approximately 25% had substantially less symptoms and better function compared to those with Current ME/CFS. Comorbidities did not differ significantly between the Current and Past ME/CFS groups.

**Conclusion:**

Further study to better understand the reasons why those in the Past ME/CFS group report no longer having the disorder is important for understanding the natural history and disease burden of ME/CFS. Studying symptomatic remissions, and the underlying physiology of improvement, could lead to identification of new disease modifying therapeutic approaches.

## Introduction

Myalgic encephalomyelitis/chronic fatigue syndrome (ME/CFS) is a persistent and disabling medical disorder [1]. People with ME/CFS experience activity limitations related to symptoms such as fatigue, post-exertional malaise (PEM), unrefreshing sleep, memory and concentration problems, orthostatic issues, and painful discomfort [2–4]. Currently, there is no known cause of ME/CFS, but it has been demonstrated to occur after acute infections [5, 6], including SARS-CoV-2 infection [7]. ME/CFS is often undiagnosed, misdiagnosed, or misattributed to other physical and mental health disorders [8]. To date, no diagnostic biomarkers have been found, and many clinicians have limited experience with ME/CFS [1, 9].

ME/CFS is diagnosed based on symptom characteristics and medical examination to identify and treat conditions that may contribute to these symptoms. Since 2015, the Institute of Medicine (National Academy of Medicine) clinical case definition [8] has been widely adopted, but other case definitions continue to be used in the field [10, 11]. Agreement over which criteria are best for clinical and research settings is not settled. Furthermore, a clinical ME/CFS diagnosis requires a medical examination with the intent of excluding other diagnoses that present with similar symptoms. It is recognized that the clinical diagnosis of ME/CFS lacks accuracy and consistency despite efforts to improve medical education about the condition. The need for integrating symptom reporting with medical evaluation to make a clinical diagnosis creates substantial difficulties in defining ME/CFS in epidemiologic studies. Self-report of health professional diagnosis of ME/CFS is frequently used as a surrogate for ME/CFS in surveillance studies, allowing for population-based estimates of the illness as well as providing insights into the lived experience of persons who report being diagnosed with ME/CFS.

The National Health Interview Survey (NHIS) is a multi-purpose health survey conducted by the National Center for Health Statistics, Centers for Disease Control and Prevention (CDC), and is the primary source of information regarding the health of the civilian non-institutionalized household population of the United States. Beginning in 2021, the NHIS included questions on prior and current diagnosis of ME/CFS. These questions enable the characterization of symptoms, comorbidity, and disability in persons with ME/CFS and those who no longer have ME/CFS. NHIS data estimates that about 3.3 million (1.3%) American adults had been diagnosed with ME/CFS and still had the condition in 2021–2022 [12]. ME/CFS affects all sociodemographic groups, and females and older adults ages 60–69 have also been reported to have a higher likelihood of reporting a diagnosis of ME/CFS [12].

ME/CFS is generally considered to be a chronic illness, with symptoms either being constant or following a relapsing-remitting pattern [13]. However, a minority report that they once had ME/CFS but no longer have it. The frequency of this reported change in status and what it means to ‘no longer have’ ME/CFS is not clear. No longer having ME/CFS could be due to recovery from ME/CFS, recognition of a new diagnosis that explains symptoms and replaces the ME/CFS diagnosis, or no longer accepting a received ME/CFS diagnosis.

Recovery from ME/CFS itself is difficult to classify, as it can be full or partial, permanent or temporary. Full recovery in ME/CFS is thought to be uncommon. A 2014 review of 22 studies found a wide range of recovery rates between 0% and 66% in both intervention and naturalistic studies. The authors point out the discrepancy, and suggest the cause could be the wide range of illness definitions and methods [13]. Additionally, a small study in 2012 found a remission rate of 80% over a 25 year period [14]. Some define recovery as a return to normal, and improvement as symptom reduction [15, 16]. Others define recovery as an acceptance of symptoms rather than a return to their premorbid functioning [17].

As the symptoms of ME/CFS are shared with those of both chronic medical and psychiatric illnesses, the potential for misdiagnosis or late recognition of an evolving disease process is substantial. Detailed medical reviews of ME/CFS patients or those referred to ME/CFS clinics report alternative diagnoses in 33–49% [18–20]. How alternative diagnoses and their treatments would lead a person to stop reporting ME/CFS as a current medical disorder are also unknown.

Data from the NHIS surveys could provide some insights into the subset of participants who report ever diagnosed with ME/CFS but not currently having ME/CFS (Past ME/CFS). We characterized the demographics, symptoms, co-morbidities, functional ability and overall health outlook of U.S. adults who report no longer having ME/CFS and those who never had ME/CFS, comparing them to those reporting currently having ME/CFS, using data from the 2021–2023 NHIS.

## Methods

Pooled data from the 2021–2023 NHIS were used for this analysis [21–23]. The NHIS provides information on health status, health-related behaviors, and healthcare access and use. The cross-sectional survey collects data continuously, with an annual data file release. Estimates from this file, after accounting for the complex survey design of NHIS, are representative of the civilian non-institutionalized population. NHIS interviews are primarily conducted in person in the respondent’s home, but due to the COVID-19 pandemic, most of the interviews in 2021 were conducted, in part, by telephone. First, a household respondent provides basic demographic information about all individuals living in the household. Next, one adult (the “Sample Adult”) and one child (the “Sample Child”) are randomly selected to be the subjects of more detailed health interviews, which include questions about family demographics. Sample Adults respond for themselves and the family unless a mental or physical condition prevents a self-response, in which case a knowledgeable adult serves as a proxy respondent. Further information regarding the NHIS and its sample design is detailed in the 2023 NHIS Survey Description Document [24]. The NHIS was approved for data collection by the NCHS Research Ethics Review Board, with verbal informed consent obtained from all respondents. This report follows the STROBE guideline for observational studies [25].

All estimates in this report meet NCHS reliability standards. Estimates with a relative standard error (standard error divided by point estimate) greater than 0.30 or a relative confidence interval (absolute confidence interval divided by point estimate) greater than 1.33 were considered unreliable and thus are not displayed [26]. For within year comparisons, non-overlapping 95% CI for two proportion estimates were considered statistically significant with alpha set at 0.05. No adjustments were made for multiple comparisons. Formal group comparisons between Current and Past ME/CFS were conducted with z-tests using Microsoft Excel version 16.107.1. Multiple logistic regression was performed on all variables, with CFS status as the dependent variable and the demographics and each individual variable as the independent variables. Only Current and Past ME/CFS were considered in these analyses, with Past ME/CFS as the reference category. Analyses were conducted using SAS 9.4.

Three demographic variables were collapsed into fewer categories for the multiple logistic regression to improve model stability. Combining groups provided stable results, but sacrificed specificity. Race was alternatively coded to include White, Black, and other (includes Asian, Native American/Alaskan Native, and Other). Cigarette Smoker was alternatively coded to include Current (includes Every Day and Some Days), Former, and Never. Residence Owned or Rental was alternatively coded to include Owned or Not Owned (includes Rented and Other Arrangement). These changes were marked in the tables where they appear.

### Subjects

**Fig. 1:**
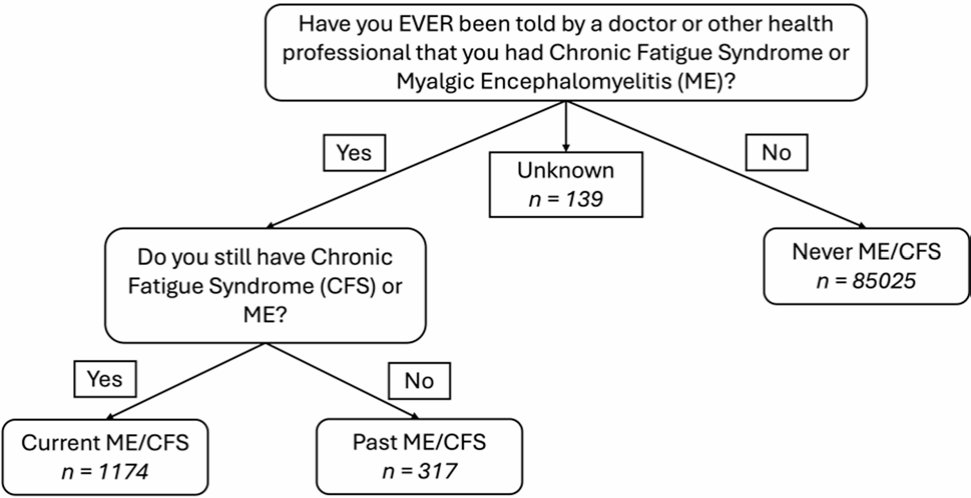
Question Pathway to Group Determination

Data from the 2021–2023 NHIS Sample Adult interview were used for this analysis; 86,655 adults participated for a weighted population size of approximately 256,000,000 adults. The Sample Adult Response Rate was 50.9% in 2021 [27], 47.7% for 2022 [28], and 47.0% for 2023 [24]. For this analysis, the outcome of interest, ME/CFS diagnosis, was categorized as follows: currently having ME/CFS, having had ME/CFS in the past, or never having had ME/CFS. Groups were determined by two questions. The first question asked, “Have you ever been told by a doctor or other health professional that you had Chronic Fatigue Syndrome (CFS) or Myalgic Encephalomyelitis (ME)?”. If the sample adult answered “Yes” to that question, they were then asked, “Do you still have Chronic Fatigue Syndrome (CFS) or ME?”. If the sample adult answered “No” to the first question, they are considered *Never ME/CFS*. If the sample adult answered “Yes” to the first and second question, they are considered *Current ME/CFS*. If the sample adult answered “No” to the second question, they are considered *Past ME/CFS*. Group determination is visualized in Fig. 1.

Flow diagram depicting how Myalgic Encephalomyelitis/Chronic Fatigue Syndrome group status was determined based on questions from the 2021–2023 NHIS. Unweighted sample sizes of each group are presented.

### Independent Variables

The independent variables were selected from the 2021–2023 NHIS question sets. While most of our analyses used variables consistently coded in all three years of the NHIS (2021, 2022, 2023), in some cases a given variable was only included in one or two NHIS years. Given our desire to describe as comprehensively as possible the characteristics of those self-reporting ME/CFS, we also examined those variables using only one or two years of the NHIS. As such, there were multiple sample sizes used during our analysis, driven by whether we were looking at 1, 2, or 3 years of data. These sample sizes varied further based on responses to funnel questions (ex: if a person reported having pain, they were asked follow up questions about the pain). For consistency, the data based on 1, 2 or 3 years of data are presented in different tables (i.e., 3-year data is presented in Table 2; 2-year data is presented in Additional File 3; and 1-year data is presented in Additional File 4. N’s for individual questions are presented in each table). The list of variables and their descriptions can be found in Additional File 1.

Participants were asked if they were ever diagnosed with a limited number of medical and psychiatric comorbidities by a health professional. Based on relevance to general health and overlapping symptoms with ME/CFS, the following comorbidities were selected: Cancer, hypertension, cardiovascular disease, prediabetes, diabetes, arthritis (including arthritis, rheumatoid arthritis, gout, lupus, or fibromyalgia), and a weakened immune system due to a health condition. The presence and severity of psychiatric comorbidity was measured in the NHIS with the following three instruments: the General Anxiety Disorder-7 [29], a self-report questionnaire used to measure anxiety symptoms, the Patient Health Questionnaire-8 [30], a tool used to assess the presence (score of ≥10 ) and severity of depression symptoms, and the Kessler 6 Serious Psychological Distress scale [31], a scale that measures psychological distress symptoms.

## Results

Unless otherwise specified, the unadjusted z-test p-value is reported for all results below. These results remained statistically significant after adjusting for sex, age, race, ethnicity, region, education, % Federal Poverty Level (FPL), marital status, ever served active duty, employed and worked last week, cigarette smoking, residence owned or bought, urban or rural county, and insurance status unless otherwise specified.

### Demographics

Of adults ≥ 18 years of age, an estimated 1.5% have been diagnosed with ME/CFS during their lifetime. Of these, an estimated 79.3%, or 3.1 million adults still had ME/CFS and 20.7%, or 820,000, no longer have ME/CFS. The most prevalent age group in Current and Past diagnoses was 60–69, and least prevalent was 40–49 (Table 1). Adults in the Current and Past ME/CFS groups were more often women (68.0% and 64.0%, respectively) compared to Never ME/CFS (51.2%). Race and ethnicity of the Current ME/CFS adults were mostly disproportionate to Never ME/CFS adults. American Indian/Alaskan Native, White, and Non-Hispanic adults were overrepresented in Current ME/CFS compared to Never ME/CFS. Asian, Other Race, and Hispanic adults were underrepresented. There were no regional differences between Current and Never ME/CFS. Current ME/CFS adults received less advanced degrees than Never ME/CFS. A higher proportion of Current ME/CFS adults reported living below the federal poverty line than Never ME/CFS adults (20.7% vs. 9.8%, respectively).

**Table 1:** Demographics of Respondents Reporting Having Current, Past, and Never Having Chronic Fatigue Syndrome or Myalgic Encephalomyelitis in the U.S. Adult General Population (2021, 2022, and 2023, National Health Interview Survey)

Demographic	Current % (CI) *N* = 1174	Past % (CI) *N* = 317	Z-Test *p*-value	Adjusted Odds Ratio	Adjusted *p*-value	Never % (CI) *N* = 85,025
Sex (% female)	68.0 (64.7–71.3)	64.0 (58.1–69.9)	N.S.	1.2 (0.8–1.7)	N.S.	51.2 (50.8–51.6)
Age						
18–39	20.1 (16.9–23.3)	20.6 (15.4–25.8)	N.S.	0.7 (0.4–1.2)	N.S.	37.6 (37.0-38.2)
40–49	13.8 (11.3–16.2)	14.2 (9.4–19.0)	N.S.	1.0 (0.5–1.7)		16.0 (15.7–16.3)
50–59	24.1 (21.1–27.0)	21.7 (16.3–27.1)	N.S.	REF		15.8 (15.5–16.1)
60–69	24.1 (21.2–26.9)	25.6 (20.1–31.2)	N.S.	0.7 (0.4–1.1)		15.3 (15.0-15.6)
70+	18.0 (15.6–20.5)	17.9 (13.3–22.5)	N.S.	0.7 (0.4–1.3)		15.3 (15.0-15.7)
Race^a^						
American Indian/Alaskan Native	3.7 (2.5-5.0)	1.8 (0.3–3.3)	N.S.	0.7 (0.4–1.4)	N.S.	1.7 (1.4–2.1)
Asian	2.6 (1.5–3.7)	3.6 (0.9–6.3)	N.S.	0.7 (0.4–1.4)		6.2 (5.7–6.7)
Black	10.8 (8.4–13.3)	4.8 (2.4–7.3)	< 0.001	1.6 (0.9-3.0)		12.3 (11.5–13.1)
White	77.8 (74.7–80.9)	81.4 (76.4–86.5)	N.S.	REF		71.7 (70.5–72.9)
Other	4.8 (3.1–6.5)	8.3 (4.1–12.5)	N.S.	0.7 (0.4–1.4)		7.9 (7.3–8.5)
Ethnicity						
Hispanic	11.5 (8.8–14.2)	15.0 (9.7–20.4)	N.S.	0.7 (0.4–1.3)	N.S.	17.3 (16.1–18.5)
Non-Hispanic	88.5 (85.8–91.2)	85.0 (79.6–90.3)	N.S.	REF		82.7 (81.5–83.9)
Region						
Northeast	14.0 (11.0-16.9)	19.2 (14.0-24.5)	N.S.	0.6 (0.4-1)	N.S.	17.5 (16.5–18.6)
Midwest	22.0 (19.0-25.1)	20.5 (15.5–25.5)	N.S.	1.0 (0.6–1.5)		20.7 (19.5–21.8)
South	42.9 (39.0-46.8)	35.6 (29.2–42.0)	N.S.	REF		38.1 (36.4–39.7)
West	21.1 (18.1–24.2)	24.7 (18.5–30.9)	N.S.	0.8 (0.5–1.2)		23.7 (22.2–25.2)
Education						
< High School	11.5 (9.3–13.8)	11.7 (7.1–16.2)	N.S.	0.7 (0.4–1.4)	< 0.05	10.1 (9.7–10.6)
High School/GED	31.2 (28.1–34.3)	22.3 (17.0-27.6)	< 0.01	REF		27.4 (26.8–28.0)
Some College/Associate	36.8 (33.6–40.1)	32.2 (26.7–37.7)	N.S.	0.9 (0.6–1.4)		28.1 (27.7–28.6)
Bachelor’s Degree	13.3 (11.1–15.4)	16.9 (12.4–21.5)	N.S.	0.7 (0.4–1.2)		21.0 (20.5–21.5)
Advanced Degrees†	6.3 (4.9–7.8)	15.6 (11.1–20.1)	< 0.001	0.4 (0.2–0.7)		12.7 (12.3–13.1)
% Federal Poverty Level						
<100	20.7 (18.0-23.4)	14.3 (9.7–18.9)	< 0.05	1.6 (0.9–2.7)	< 0.05	9.8 (9.3–10.2)
100–199	26.2 (23.3–29.2)	16.2 (11.4–21.0)	< 0.001	1.9 (1.2-3.0)		17.7 (17.2–18.1)
200+	53.1 (49.5–56.6)	69.5 (63.6–75.4)	< 0.0001	REF		72.6 (71.8–73.4)
Marital Status						
Single/Never Married	22.8 (19.6–26.0)	12.8 (8.2–17.3)	< 0.001	2.5 (1.5–4.3)	< 0.05	28.8 (28.3–29.3)
Married	42.2 (38.9–45.5)	54.7 (48.2–61.2)	< 0.001	REF		49.7 (49.1–50.3)
Divorced	19.3 (16.8–21.9)	19.9 (15.3–24.6)	N.S.	0.9 (0.6–1.5)		10.2 (10.0-10.5)
Separated	2.0 (1.1–2.9)	2.3 (0.6-4.0)	N.S.	0.8 (0.3–2.1)		1.3 (1.2–1.4)
Widowed	9.4 (7.6–11.1)	8.0 (5.2–10.7)	N.S.	0.9 (0.6–1.5)		5.9 (5.7-6.0)
Cigarette Smoker^a^						
Every Day	17.1 (14.5–19.6)	14.4 (9.9–18.9)	N.S.	0.9 (0.6–1.5)	N.S.	8.1 (7.8–8.4)
Some Days	4.1 (2.8–5.4)	3.8 (1.4–6.3)	N.S.	0.9 (0.6–1.5)		2.7 (2.5–2.8)
Former	29.0 (25.8–32.1)	31.3 (25.0-37.5)	N.S.	0.9 (0.6–1.3)		21.8 (21.3–22.2)
Never	47.0 (43.5–50.6)	49.4 (43.0-55.8)	N.S.	REF		64.3 (63.7–64.9)
Insurance						
Insured	94.2 (92.4–95.9)	90.7 (86.2–95.1)	N.S.	REF	N.S.	90.2 (89.7–90.6)
Uninsured	5.4 (3.7–7.1)	9.3 (4.9–13.8)	N.S.	0.5 (0.3–1.1)		9.4 (9.0-9.9)

All statistical testing was conducted between Current ME/CFS and Past ME/CFS groups

†:Advanced Degrees: Master’s and Doctoral Degrees

^a^:Some groups combined for adjusted analyses (see Methods)

There were several statistically significant demographic differences by z-tests between adults with Current ME/CFS and Past ME/CFS (Table 1, Additional File 2). Compared to Current ME/CFS adults, Past ME/CFS adults more frequently received Advanced Degrees (*p* < 0.001), had family income above 200% the Federal Poverty Level (*p* < 0.0001), were more frequently married (*p* < 0.001), and worked in the last week (*p* < 0.001). Current ME/CFS adults were more frequently black (*p* < 0.001), completed HS/GED more frequently (*p* < 0.01), lived at < 100% or 100–199% FPL (*p* < 0.05, *p* < 0.001, respectively) more frequently, were single/never married more frequently (*p* < 0.001), and did not work last week more frequently (*p* < 0.01) compared to Past ME/CFS adults. Differences in sex, age, race, ethnicity, cigarette smoking, insurance status, ever serving active duty, living in an urban or rural county, residence owned or rental, and region of residence were not statistically significant after multivariate adjustment.

### Symptoms

Both Current and Past ME/CFS adults reported statistically significant greater symptoms and impairment compared to those who never had ME/CFS (Table 2, Additional Files 3–4).

**Table 2:** Combined Prevalence and Severity of Symptoms for Questions Asked in 2021, 2022 and 2023

Variable	Category	Current (% CI)	Past (% CI)	Z-Test *p*-value	Never (% CI)
Cognitive		*N* = 1174	*N* = 317		*N* = 85,025
Difficulty communicating	A lot/cannot do at all	3.7 (2.4–5.1)	0.9 (0.0-1.9)	< 0.001*	0.8 (0.7–0.9)
	No difficulty	79.4 (76.4–82.3)	88.8 (85.1–92.5)	< 0.0001*	94.7 (94.5–94.9)
Difficulty remembering/Concentrating	A lot/cannot do at all	18.8 (15.9–21.6)	9.3 (5.5–13.1)	< 0.0001*	2.5 (2.3–2.6)
	No difficulty	32.8 (29.7–35.9)	53.2 (46.8–59.6)	< 0.0001*	80.2 (79.8–80.7)
		*N* = 698	*N* = 134		*N* = 15,292
How often difficulty remembering?	Always	16.6 (13.2–20.0)	11.2 (4.6–17.9)	N.S.	8.7 (8.1–9.2)
	Sometimes	50.6 (46.0-55.2)	61.7 (51.9–71.4)	< 0.05	72.2 (71.3–73.2)
Amount of things difficult to remember?	Almost everything	9.9 (7.2–12.6)	6.3 (2.0-10.5)	N.S.	4.6 (4.2–5.1)
	A few things	63.4 (58.7–68.1)	80.2 (73.3–87.1)	< 0.0001	83.4 (82.6–84.2)
Psychiatric		*N* = 1174	*N* = 317		*N* = 85,025
How often feel worried, nervous, or anxious	Daily	46.4 (42.9–50.0)	32.3 (26.4–38.3)	< 0.0001*	12.8 (12.5–13.1)
	Never	9.0 (7.2–10.9)	14.7 (10.2–19.3)	< 0.05*	28.0 (27.4–28.6)
How often depressed	Daily	25.8 (22.8–28.9)	10.6 (7.1–14.1)	< 0.0001*	3.7 (3.6–3.9)
	Never	17.3 (14.8–19.9)	27.4 (22.2–32.6)	< 0.001*	52.4 (51.8–53.0)

All statistical testing was conducted between Current ME/CFS and Past ME/CFS groups

*: p<0.05 after multivariate adjustment for sex, age, race, ethnicity, region, education, % Federal Poverty Level (FPL), marital status, ever served active duty, employed and worked last week, cigarette smoking, residence owned or bought, urban or rural county, and insurance status

### Symptoms Queried in 2021, 2022, and 2023 Combined

Symptom data gathered from all three years (2021, 2022, and 2023) fell into the cognitive and psychiatric categories (Table 2). Cognitive problems were frequent in ME/CFS groups compared to Never ME/CFS. Difficulty communicating was infrequent but more often reported in Current ME/CFS than Past ME/CFS adults (*p* < 0.001), while no difficulty communicating was reported more frequent in Past ME/CFS (*p* < 0.0001). Only a third of Current ME/CFS adults and about one-half of Past ME/CFS adults had no difficulties remembering/concentrating (*p* < 0.0001). Conversely, Current ME/CFS adults had a larger amount of difficulty remembering/concentrating (*p* < 0.0001). The frequency of remembering difficulties and amount of things difficult to remember were not different after adjustment.

Current ME/CFS adults also reported more psychiatric symptoms than Past ME/CFS adults. Significantly more Current ME/CFS reported daily symptoms of anxiety compared with Past ME/CFS (*p* < 0.0001). Daily symptoms of depression were also more frequently reported by Current ME/CFS than Past ME/CFS (*p* < 0.0001). Past ME/CFS reported never feeling anxious or depressed more frequently than Current ME/CFS (*p* < 0.05 and *p* < 0.001, respectively).

### Symptoms Queried in 2021 and 2023

In 2021 and 2023, the NHIS asked about pain symptoms (Additional File 3). As the NHIS did not query these symptoms in all three years considered in this study, these are best considered exploratory outcomes. Both Current and Past ME/CFS adults reported statistically significant greater symptoms and impairment compared to those who Never had ME/CFS.

Current ME/CFS adults experienced pain ‘most/every day’ over a 3-month period significantly more often than Past ME/CFS adults (*p* < 0.0001). A pain frequency of ‘never’ was reported rarely by Current ME/CFS adults and infrequently by Past ME/CFS adults (*p* < 0.01). Pain that was ‘a little bothersome’ (*p* < 0.05) was significantly different between Current and Past ME/CFS adults while pain that was ‘a lot bothersome’ was not. Pain amount was not significant after multivariate adjustment.

### Symptoms Queried in Individual Years

Some variables were only queried in one year (Additional File 4). Never ME/CFS reported statistically significantly less symptoms and impairment than both Current and Past ME/CFS. The 2021 NHIS administered the K6 Serious Psychological Distress scale. A quarter of Current ME/CFS met criteria for serious psychological distress, about three times that observed in Past ME/CFS (*p* < 0.0001), but this difference was not statistically significant after multivariate adjustment.

Fatigue, sleep, and the GAD-7 and PHQ-8 were queried in the 2022 NHIS. Current ME/CFS adults reported more fatigue symptoms than Past ME/CFS adults. Most Current ME/CFS adults were tired most days or every day during a three-month period compared to about one-third of Past ME/CFS adults (*p* < 0.0001). When tired, Current ME/CFS reported it lasted all of the day more than Past ME/CFS (*p* < 0.05), but Past ME/CFS reported tiredness lasting ‘some of the day’ more frequently than Current ME/CFS adults (*p* < 0.0001). Current ME/CFS reported the level of tiredness as ‘a lot’ more frequently than Past ME/CFS (*p* < 0.01). Almost two times the amount of Current ME/CFS adults had trouble falling asleep ‘most or every day’ than Past ME/CFS adults (*p* < 0.001). Current ME/CFS adults also never felt rested at a higher frequency than Past ME/CFS adults (*p* < 0.0001).

There was a difference of approximately 16% in those Current ME/CFS adults who report never having trouble staying asleep compared with Past ME/CFS adults (*p* < 0.01). Compared with Past ME/CFS adults, Current ME/CFS adults reported feeling rested “most or every day” significantly less than Past ME/CFS (*p* < 0.0001). Past ME/CFS reported never having trouble falling asleep significantly more than Current ME/CFS (*p* < 0.05), but no difference was noted after multivariate adjustment.

The number of Current ME/CFS adults that met criteria for “Severe Depression” symptoms on the PHQ-8 was significantly higher compared to Past ME/CFS adults (*p* < 0.001). Conversely, more Past ME/CFS adults met criteria for “None/Minimal Depression” than Current ME/CFS (*p* < 0.0001). There was no significant difference in “Severe Anxiety” or “None/Minimal Anxiety” after multivariate adjustment.

The 2023 NHIS asked about balance and dizziness problems. These were more frequent in Current ME/CFS adults than Past ME/CFS adults (*p* < 0.001). Approximately one-third of Current ME/CFS adults and approximately half of Past ME/CFS adults did not experience balance or dizziness problems (*p* < 0.001).

### Comorbidities

Of the comorbidities reviewed (Table 3), the adjusted prevalence of arthritis and related conditions, Weakened Immune System Due to a Health Condition (WISDHC), and prediabetes were increased in Current ME/CFS compared with Past ME/CFS. The prevalence of cancer, coronary heart disease, diabetes, and hypertension were not different between the groups after adjustment for confounding variables. Comorbidities were significantly less prevalent in Never ME/CFS compared to Current and Past ME/CFS.

**Table 3:** Prevalence of Comorbidities

Variable	Current % (CI)	Past % (CI)	Z-Test *p*-value	Never % (CI)
Arthritis^a^	67.1 (63.9–70.4)	56.0 (49.5–62.4)	< 0.01*	20.6 (20.2–21.1)
Weakened immune system due to a health condition	30.9 (27.8–34.0)	16.1 (11.6–20.5)	< 0.0001*	4.3 (4.1–4.5)
Cancer	17.0 (14.6–19.4)	16.1 (11.8–20.5)	N.S.	9.6 (9.3–9.8)
Coronary heart disease	15.4 (13.2–17.7)	10.3 (6.9–13.6)	< 0.05	4.7 (4.6–4.9)
Diabetes	22.2 (19.2–25.1)	17.5 (12.9–22.1)	N.S.	9.5 (9.2–9.7)
Prediabetes	16.1 (13.5–18.7)	12.7 (8.8–16.6)	N.S.*	8.7 (8.4–8.9)
Hypertension	43.8 (40.3–47.2)	35.0 (29.2–40.8)	< 0.05	21.7 (21.2–22.1)

All statistical testing was conducted between Current ME/CFS and Past ME/CFS groups

*: p<0.05 after multivariate adjustment for sex, age, race, ethnicity, region, education, % Federal Poverty Level (FPL), marital status, ever served active duty, employed and worked last week, cigarette smoking, residence owned or bought, urban or rural county, and insurance status

^a^:  Arthritis, rheumatoid arthritis, gout, lupus, or fibromyalgia

### Disability

Both Current and Past ME/CFS reported significantly more disability indicators than Never ME/CFS (Table 4). Past ME/CFS adults reported experiencing no difficulties with daily activities more frequently than Current ME/CFS adults. The frequency of Current ME/CFS adults reporting no difficulty in doing errands alone was about 20% less than Past ME/CFS adults (*p* < 0.0001). Similar proportions reported having no difficulty participating in social activities, work being limited due to a health problem, not working due to health reasons, not receiving home healthcare, and in not meeting the Disability Indicator threshold.

**Table 4:** Prevalence of Disability

Variable	Category	Current % (CI)	Past % (CI)	Z-Test *p*-value	Never % (CI)
Difficulty doing errands alone	Cannot do at all	12.0 (9.7–14.2)	3.9 (1.7–6.2)	< 0.0001*	2.6 (2.4–2.7)
	No difficulty	54.4 (51.0-57.8)	75.6 (70.1–81.2)	< 0.0001*	91.7 (91.4–91.9)
Difficulty participating in social activities	Lots	27.3 (24.1–30.6)	9.6 (5.9–13.3)	< 0.0001*	3.9 (3.8–4.1)
	No difficulty	46.6 (43.3–49.8)	69.1 (63.2–75.0)	< 0.0001*	89.6 (89.3–89.9)
Work limited due to health problem	Yes	72.6 (69.6–75.6)	47.6 (41.3–54.0)	< 0.0001*	18.3 (17.9–18.8)
	No	27.4 (24.4–30.4)	52.3 (45.9–58.7)	< 0.0001*	81.6 (81.1–82.0)
Main reason not working^#^	Health reasons/disabled	50.6 (46.6–54.7)	33.6 (24.9–42.3)	< 0.001	14.9 (14.3–15.5)
Received home health care	Yes	13.9 (11.5–16.4)	7.5 (4.5–10.6)	< 0.01*	3.5 (3.4–3.6)
	No	84.5 (81.9–87.1)	91.7 (88.5–95.0)	< 0.001*	94.8 (94.6–95.0)
Disability Indicator	Yes	47.6 (44.1–51.2)	27.3 (21.6–33.1)	< 0.0001*	8.6 (8.3–8.8)
	No	52.4 (48.8–55.9)	72.7 (66.9–78.4)	< 0.0001*	91.4 (91.2–91.7)

All statistical testing was conducted between Current ME/CFS and Past ME/CFS groups

*: p<0.05 after multivariate adjustment for sex, age, race, ethnicity, region, education, % Federal Poverty Level (FPL), marital status, ever served active duty, employed and worked last week, cigarette smoking, residence owned or bought, urban or rural county, and insurance status

^#^: Multivariate adjustment not performed on this question

The percentage of Current ME/CFS adults who could not do errands alone was three times that among Past ME/CFS adults (*p* < 0.0001). Similarly, a higher percentage of Current ME/CFS adults had a lot of difficulty doing social activities compared to Past ME/CFS adults (*p* < 0.0001). The majority of Current ME/CFS found that their ability to work was limited due to a health problem compared to nearly half of Past ME/CFS adults (*p* < 0.0001). Half of Current ME/CFS adults reported they were unable to work due to health reasons compared to a third of Past ME/CFS adults (*p* < 0.001). The percentage of Current ME/CFS adults who received care at home was twice that among Past ME/CFS adults (*p* < 0.01). Additionally, almost half of Current ME/CFS adults met the Washington Group Short Set Composite Disability Indicator compared to about a quarter of Past ME/CFS adults (*p* < 0.0001).

### Health Status

Participants were also asked how they ranked their health on a scale of excellent, very good, good, fair, or poor (Table 5). Both Current and Past ME/CFS reported significantly worse general health status than Never ME/CFS.

**Table 5:** General Health Status

Health Status	Current % (CI)	Past % (CI)	Z-Test *p*-values	Never % (CI)
Excellent	1.7 (0.7–2.6)	7.2 (4.1–10.4)	N.S.*	23.3 (22.8–23.7)
Very good	7.5 (5.8–9.2)	25.0 (19.3–30.7)	N.S.*	34.8 (34.3–35.3)
Good	24.6 (21.6–27.5)	26.9 (21.0-32.7)	*p* < 0.0001*	28.7 (28.3–29.2)
Fair	37.3 (33.9–40.6)	27.6 (21.7–33.5)	*p* < 0.0001*	10.5 (10.2–10.8)
Poor	29.0 (25.8–32.3)	13.4 (9.0-17.8)	*p* < 0.0001*	2.7 (2.6–2.9)

All statistical testing was conducted between Current ME/CFS and Past ME/CFS groups

*: p<0.05 after multivariate adjustment for sex, age, race, ethnicity, region, education, % Federal Poverty Level (FPL), marital status, ever served active duty, employed and worked last week, cigarette smoking, residence owned or bought, urban or rural county, and insurance status

Excellent, Very Good, or Good was reported less frequently by Current ME/CFS adults (34%) compared with Past ME/CFS adults (58%), a difference of 24% in positive health outlook. Current ME/CFS adults infrequently rated their health as Excellent and Very Good (9.2%), while nearly one-third of Past ME/CFS adults (32.2%) rated their health to be this positive. Among Current ME/CFS adults, 66.4% reported their health status as either Fair or Poor, compared to 40.9% of Past ME/CFS adults (*p* < 0.0001).

## Discussion

These data are the first to characterize people who report having a past but not current diagnosis of ME/CFS. Of United States adults reporting having had ME/CFS at some point in time, one-fifth report that they no longer do. The NHIS data provide interesting observations about the nature of self-reported remission from ME/CFS.

First, a substantial proportion of adults reporting no longer having ME/CFS still report high levels of impairment. Poor and poor to fair health was reported by 15.6% and 41% of adults with Past ME/CFS, respectively. Nearly half report that their health limits their ability to work and a third report that health is why they are not working. More than a third feel tired most of the time, 20% never feel rested, and nearly half have pain most of the time. For these 40–50% of adults with Past ME/CFS, estimates of symptoms and function are not much different than adults with Current ME/CFS.

Second, the data does not support that receiving a new diagnosis was a major reason for self-reporting ME/CFS remission. Comorbidity reporting is generally higher in Current ME/CFS adults, which does not suggest diagnostic misattribution. Unfortunately, the NHIS does not include some health conditions germane to ME/CFS, such as Multiple Sclerosis and Post-Transplant Lymphoproliferative Disorder. If the ME/CFS diagnosis was later found to be the result of a different diagnosis, those co-morbidities should be more frequent in adults with past ME/CFS.

Third, the data demonstrates that about 25% of adults with past ME/CFS have current measures of symptoms, function, and health status that suggest they currently do not have ME/CFS symptoms. Past ME/CFS report never being tired (10%), being pain free (10%), feeling rested most days (24.6%), not having difficulties with remembering and concentrating (20%), being minimally depressed (23%), not having balance or dizziness issues (21.8%), and not being disabled (20.3%). The past ME/CFS group view their health as excellent or very good (32.2%) more frequently than the Current ME/CFS (9.2%). While this cross-sectional survey cannot demonstrate causality, these overlapping estimates of comparatively decreased symptom burden and improved functional ability in persons reporting past ME/CFS are suggestive of recovery trajectories. Whether this ‘recovery’ reflects a permanent change or just a current remission of symptoms in a disorder known to fluctuate cannot be determined in this data. However, at best it appears that only a quarter of self-reported ME/CFS remission can be attributed to a clinical picture consistent with permanent or temporary recovery.

A final possibility for self-reported ME/CFS remission can simply be that the individual no longer endorses the diagnosis despite ongoing symptoms. This was not directly queried by the NHIS. Future surveys of the topic would benefit from developing questions to explore why a person self-reports ME/CFS remission.

There are some other technical issues to consider when interpreting this data. The characteristics of ME/CFS in the general U.S. population as measured by the NHIS differ significantly from those observed in clinical research studies. While systematic review of clinical trials would suggest that ME/CFS is a disorder of middle age [32], the greatest percentages of reported ME/CFS in the NHIS were in older age groups, with 42% of respondents being older than 60. ME/CFS was more prevalent in women than men, but the 2:1 ratio observed is substantially less than seen in clinical trials [32]. Adults with ME/CFS in the NHIS tended to receive less education than Never ME/CFS comparators. While illness presenting during school and college ages could interfere with educational attainment, the NHIS results do not align with a Norwegian study that suggests that higher education is associated with ME/CFS risk [33]. Considered together, these data suggest that many of the individual research studies in the ME/CFS literature may not generalizable to the greater population of ME/CFS.

Some of this may be related to the limitations of epidemiologic study of ME/CFS. Self-report of a health professional’s diagnosis is not equivalent to meeting ME/CFS diagnostic criteria [34–36]. Post-exertional malaise and dysautonomia, key symptoms of ME/CFS and clinical diagnostic criteria, are not directly queried by the NHIS. All data collected was self-reported and subject to recall biases. Medical record verification of diagnoses was not performed. Rather, the NHIS survey data represents how each participant views their health and the diagnoses they recall receiving.

However, the NHIS survey data reflects the prevalence of ME/CFS as recognized by communities across the US and therefore reflects the diagnostic uncertainties such patients face. Clinician recognition of ME/CFS and other syndromes is variable and when syndromes are recognized, it is unlikely that published criteria are carefully applied, resulting in lack of standardization [37]. The high levels of reporting known ME/CFS symptoms suggest that those reporting ME/CFS diagnoses have health problems that approximate those used in diagnostic criteria.

Some questions were not asked in each survey, limiting the number of responses. We note the questions in which this occurred and indicate these as exploratory analyses. The NHIS information includes a limited number of other health conditions and timing of diagnoses are included. Lastly, the years of interest of this study correspond to the SARS-CoV-2 pandemic, which may have impacted reporting of both Current and Past ME/CFS.

In conclusion, approximately 20% of adults who have had ME/CFS report no longer having the disorder. About half of adults with past ME/CFS continue to report severe levels of symptoms and a quarter of them report symptoms, functioning, and general health suggestive of a symptomatic recovery. Comorbid health conditions did not appear to impact self-reported remission. Further studies should focus on identifying the factors that contribute to self-reported remission in ME/CFS patients, as this could inform treatment strategies and improve patient outcomes.

## Data Availability

The publicly-available data used in this article can be accessed for free at the National Center for Health Statistics: [NHIS Questionnaires, Datasets, and Documentation | National Health Interview Survey | CDC](https:/www.cdc.gov/nchs/nhis/documentation/index.html).
